# The Importance of Chemical and Microscopical Examination of the Urine*Read before Medical Association of Georgia, at Brunswick, April 17, 1890.

**Published:** 1890-05

**Authors:** Howard J. Williams

**Affiliations:** Macon, Ga.


### Southern Medical Record.

A MONTHLY JOURNAL OF MEDICINE AND SURGERY.

VOL. XX. ATLANTA, GA., MAY, 1890. NO. 5.

### Original Articles.

### THE IMPORTANCE OF CHEMICAL AND MICROSCOPICAL EXAMINATION OF THE URINE.*

BY HOWARD J. WILLIAMS, A. M., M. D., MACON, GA.

The frequent newspaper reports of sudden death from "unknown or natural causes," and the large number of deaths in mortuary reports recorded as due to heart failure, coma, convulsions, dropsy, apoplexy, etc., arrests the attention of the thoughtful, and suggests the inquiry—what are the pathological changes primarily causing these fatal symptoms? We can, with safety, attribute a very large number of these deaths to renal disease, the presence of which was never recognized. Renal changes are attended by symptoms common to other disorders, and, if their nature is not recognized, they are easily mistaken. The apparently increasing spread of Bright's disease throughout the country, its latency, and the insidiousness of the early symptoms, is such, that it behooves the careful diagnostician to be constantly on the alert.

Life insurance corporations, appreciating the value of early recognition of the signs of these diseases, no longer request examinations of the urine of applicants for large policies only, but now require careful urinary analysis in all applications.

The correct diagnosis of Bright's disease is based chiefly

*Read before Medical Association of Georgia, at Brunswick, April 17, 1890.

upon the careful examination of the urine, both chemical and microscopical. What then are some of the requirements for careful urinary analysis, and what is their value in correct recognition of kidney lesions?

Stated broadly, the urine must be repeatedly examined to ascertain its quantity, its specific gravity, the presence or abscence of tube casts, and, lastly, the estimation of the quantity of urea excreted. Frequently repeated examinations in doubtful cases are necessary since variations in the quantity, specific gravity, and presence or absence of albumin and casts are liable to take place from day to day, in all cases, and an examination during any temporary change might lead to incorrect diagnosis. While it is generally true that signs of the disease may be found at some time in all cases, yet persons have been known to have chronic lesions of the kidneys for years without the presence of signs and symptoms, and, after death from accident or other disease, chronic renal changes were found by post-mortem examinations. Furthermore, symptoms of a latent renal disease may be brought to light by some intercurrent malady.

The daily quantity of urine should be known, since an increased or diminished quantity is indicative of certain pathological changes. But the quantity, either increased or diminished, cannot alone be considered diagnostic, since changes may be temporarily brought about by processes not pathological. Changes in quantity to mean anything, must persist in the urine of repeated examinations, and must be attended by characteristic changes in color, specific gravity, and presence of albumin or casts.

The specific gravity is sometimes regarded as diagnostic of kidney lesions; but its value is doubtful, unless it is persistent. A temporary increase of the solids or diminution of the watery elements of the urine might give a high specific gravity that could be easily misconstrued, if the diagnosis rested simply upon one examination. The average of many examinations, in conjunction with other signs, must alone be of value. So important, however, is this sign, that life insurance companies will not accept risks having a continuously high or low specific gravity.

The presence of albumin in the urine is an important consideration in the diagnosis of renal disease. For a long time after its existence in kidney changes was discovered, its presence was regarded as a fatal omen. But more recent investigations have shown that it is not pathogonomic, and it is now no longer dreaded. It is found present in other conditions, and under circumstances not fatal. It is now known to be present in health, and in morbid processes not renal, as cardiac disease, irritations of the central nervous system, infectious fevers, and some local disorders, as of the bladder and urethra, and in pregnancy. Its presence in these disorders cannot be taken as evidence of associated renal lesions. Though generally present at some time during Bright's disease, it is absent sufficiently often to make it of doubtful diagnostic value, and its absence from the urine is not a guarantee that renal changes are not present. It is often present, and yet escapes detection by the usual tests. Recently a specimen failing to respond to heat and nitric acid, was referred to Dr. McHatton and myself for further examination, and, with other re-agents, we found albumin. In doubtful cases therefore, repeated examinations should be made; and not one or two, but several re-agents should be employed. As pathogonomic of Bright's disease, and in the differentiation of its various forms, albumin alone is not sufficient; to be of value, it must be associated with other signs.

Additional evidence of renal disease is obtained by the microscopical examination of the urine, and the detection of tube casts or cylinders. These casts are of clinical importance, as they come from the depths of the uriniferous canals of kidneys whose functions are defective. Their presence continuously in the urine is the best kind of evidence of renal changes, since they can be found when albumin cannot be detected, and their characteristic appearances may be useful in the differentiation of the various types of the disease. Like albumin, they may be temporarily absent from the urine, and one examination at this time may lead to false conclusions. Again, they are occasionally found in health, and under circumstances not dependent on renal changes; hence, to be of value, they must be found in repeated examinations. Bright's disease has been known to exist in an individual without the presence of casts; therefore, their absence cannot be infallible evidence of freedom from disease.

While albumin and casts are abnormal products eliminated by the kidneys in Bright's disease, urea is a normal ingredient of the urine, which is found to be excreted in diminished quantities.

This diminished quantity has, hitherto, been of clinical interest simply as evidence that a certain quantity of urea is retained in the blood, producing, directly or indirectly, uræmic poisoning. Recently, however, it is assuming importance with reference to the recognition of chronic renal disease. Much is being written on this question, and so high an authority as Dr. T. Grainger Stewart employs it as a diagnostic test for kidney lesions.

Dr. C. S. Bond, of Richmond, Ind., has a paper in the *American Journal of Medical Sciences*, January, 1890, on the subject. He bases his conclusions upon the repeated examinations of fifty cases, and his observations cover a period of more than five years. This diminished urea is found in urine containing albumin and casts; and it is also found in the urine of patients having the outward symptoms of renal disease, long before albumin and casts are discovered. If this latter fact will hold good in all cases, examination for the quantity of urea may be used as a test for the early stages of Bright's disease. Like the other signs of kidney changes, it is said to be only of value in the presence of other symptoms, and repeated examinations are necessary to establish the validity of its evidence.

It would seem, from the above considerations, that all these signs of renal diseases are defective, and, taken separately, unreliable. This, in a measure, is true; yet, upon the association of several, during many observations, rests their value. Upon these several signs, then, depends the almost absolute recognition of a disease, whose pathology is far-reaching; not simply a lesion of the kidney only, but, as revealed by recent investigations, an involvment of other important tissues. In the light of these recent discoveries in pathology, may we not hope for other signs of greater value, and readier methods of detecting the earlier changes of kidney diseases?

Having considered these diagnostic signs found in the urine, let us turn to some of the general symptoms and complications calling for urinary analysis. As before stated, the disease is so insidious, and many symptoms so easily attributed to other diseases, that renal changes may exist, and be far advanced, before they are recognized.

The nervous symptoms of Bright's disease demand careful consideration. Coma and convulsions are common terminations of the disease. A fatal uræmic coma may strike down a person of unknown history, and, if there is no urinary analysis, the trouble may be mistaken for apoplexy or for narcotic poison. So irritable is the nervous system in renal disease, that, like the highly organized system of childhood, the slightest disturbance of equilibrium of functions is sufficient to bring on convulsions. Persistent neuralgic and chronic rheumatic affections are frequently expressions of chronic renal lesions; no dropsy or other outward symptoms being present to call attention to the kidneys. Such cases proving rebellious to treatment, suspicion is aroused, and examinations of the urine may disclose their true source.

Oedema of the Lungs, Chronic Bronchitis, and Asthma, of renal origin, have no characteristic symptoms, except those revealed by the urine. Treatment directed to these disorders, without consideration of the kidney affection, can only result in failure. The true origin of the following case was determined by examination of the urine, after months of suffering from erroneous diagnosis, and misguided therapeutics, owing to the neglect of urinalysis.

Mrs. B., white, aged 47 years, consulted me in February, 1889, for a distressing cough, which had been pronounced a Chronic Bronchitis, and so treated, without relief, for several months. She suffered with extreme dyspnœa, attended by cyanotic lips, and inability to lie down. She expectorated large quantities of white frothy mucus, stained with pus and blood. Large and small bronchial rales could be heard throughout the chest. The cardiac area was enlarged; the heart over-active; the pulse small, feeble and rapid. She had constant headache; occasional attacks of nausea and vomiting, and some oedema of the ankles.

The daily quantity of urine was diminished to twelve or twenty ounces; dark colored; specific gravity 1018 to 1022; only a very slight trace of albumin could be detected by the most careful testing; while hyaline and granular casts, and, at times, blood corpuscles were present. Upon close inquiry, she gave a history of acute dropsy, followed by apparent recovery, three years previous. She has been under my observation for fourteen months; the bronchitis is relieved; her general condition is improved; yet she has had several mild uraemic attacks, Her family know her condition; are prepared to meet her uraemic symptoms, and will not be surprised by an unexpected termination of her disease.

In affections of the heart, urinalysis is of great value, both in diagnosis and prognosis. No two classes of disease are more intimately related than those of the heart and of the kidneys; therefore, the study of the one is essential in the presence of the other. This association is particularly marked between cardaic hypertrophy and chronic interstitial nephritis, and, in a given case, with symptoms suggestive of both disorders, it may become necessary to decide whether the disease is the former with attendant renal congestion, or the latter with heart trouble, due to changes in the blood and blood-vessels. Albumin is found in both conditions, but tube casts, rarely present in purely cardaic disease, are nearly always found in contracted kidneys. These cases of contracted kidneys with hypertrophied heart, usually die suddenly with uraemic symptoms; the detection of albumin or casts may give warning in time to delay, by judicious therapeutics, the fatal day. In a feeble heart with disturbed renal functions, the detection of albumin with casts is a fatal omen, and gives warning of a sudden ending. In the following case, urinalysis gave this warning, and its sudden fatal issue did not find her friends unprepared.

Mrs. S., white, age 70 years, had a history of an attack of angina pectoris 12 years ago. Since then she has had to take, at times, digitalis for palpitation. When called to see her, June 25, 1889, she had been suffering for several days with dizziness and a full feeling in the head; she saw specks floating before her eyes and heard peculiar noises, and she complained of cardiac uneasiness. The pulse was feeble, easily compressed, and remittent. The cardiac area was enlarged, and its impulse feeble. No murmurs were heard on ausculta-tion, but the cardiac sounds were feeble and intermittent, though not irregular. She had some slight œdema of the ankles. The urine had recently increased in quantity—the daily amount was two quarts. Specific gravity 1020; albumin largely deposited, hyaline and granular casts present. Under treatment appropriative to kidney disease, she improved up to August 5th, though albumin and casts remained largely present. On this day, while engaged with her household duties, she was suddenly seized with severe pain in the back of her head, followed immediately by unconsciousness and stertorous breathing. After an hour consciousness partially returned. As soon as the urine could be collected it was measured, and amounted to 10 ounces in twenty-four hours; specific gravity 1016; albumin abundantly deposited; tube casts present. Vomiting, headache, irregular action of the heart, attacks of blindness and hallucinations of hearing continued some days; her condition then slightly improved. September 1st, after a night of restlessness, headache and vomiting, she passed into a quiet sleep. Her attendant went out of the room for a few moments, and, returning, found that she was dead.

Of all the complications of Bright's disease, those of the digestive organs are the most commonly overlooked. Chronic catarrh of the stomach, with nausea and vomiting; catarrh of the intestines, with profuse diarrhœa and functional disorders of the liver, are found associated with this disease. The headaches, nausea and vomiting, and so-called torpidity are often ignorantly attributed to a disturbance of the hepatic functions, popularly known as "biliousness." Even the more violent forms of uraemic poisoning, coma, or convulsions, have been thus mistaken. Two or three years ago Dr. McHatton reported a case of the latter character in the Atlanta *Medical and Surgical Journal*, and called attention to the frequent erroneous interpretation of this co-called "biliousness." To complete the history of this case, and to show to what fatal ends this error may lead, I will state that, shortly after this report appeared, the patient moved away from Macon, and, notwithstanding the most positive assurance that the disorder was uraemic, and the patient so instructed to inform his next medical adviser, he died in one of these uraemic attacks, his death being attributed to heart disease. I have at present under my charge one of these "bilious" individuals, whose urine has a constant specific gravity of 1010, and contains a trace of albumin and tube casts. He is wedded to his "biliousness," because his first attendant so named his disorder. Such mistakes will occur less frequently when urinalysis becomes more generally employed in these simple indispositions.

These sudden attacks of vomiting and diarrhœa are frequently beneficial. The blood, charged with urea or other poisonous matter, irritates the stomach and bowels, provoking vomiting or diarrhœa, which in time drains the poisonous fluids from the system.

These attacks, at the same time, are prognostic; they are often the forerunners of grave uraemic symptoms. The knowledge gained in such conditions, by examining the urine, is invaluable, indicating measures for aiding the elimination of the poison. It would be criminal, if, knowing the character of such attacks, remedies to arrest these symptoms were not employed.

Recently a man of intemperate habits had a severe attack of vomiting; a hypodermic injection of morphine relieved the vomiting; a night's rest was obtained, but the next morning, on attempting to cross the room, he fell, and, in two hours, died comatose. Examination of his urine, no doubt, would have revealed evidences that he was suffering with uraemic poisoning.

This next case is of peculiar interest in this connection, illustrating the latency of renal disease. The possible uraemic origin of some diarrhœas; the mistake of arresting such a discharge, terminating in convulsions and coma; and suggesting the possible value of urinalysis in spite of a supposed simple etiology.

Mr. E., white, a large, healthy looking man, of temperate habits, had not been ill for fifteen years until this attack, which began as a mild but rebellions diarrhœa three weeks before my first visit. I saw him July 15, 1889, in the absence of his family physician, for what he regarded as a relapse. He had frequent watery stools, great thirst, occasional vomiting, headache and sleeplessness, but no fever. His disorder was attributed by his physician to eating over-ripe fruit. As others in his family were simultaneously affected with a similar disorder, the conclusion was plausible. Under ordinary treatment his diarrhœa was checked, and I dismissed him at my third visit. His urine had never been tested.

July 23d I was again called to see him. He was restless, had not slept two out of seventy-two hours, had nausea and an unquenchable thirst. He would drink, his wife said, three gallons of water during the day and night. He passed large quantities of urine, which I requested to be measured, and a large specimen furnished me the next morning. From 5 p. m· of the day of my visit to 5 p. m. the next day, he had passed four quarts of colorless urine. The specific gravity was 1002, and contained neither albumin or sugar. The urine was allowed to settle several hours; tube casts were then found with the microscope. I did not see Mr. E. during the second morning; but at 5 p. m. I was sent for urgently to see him, as he was unconscious and thought to be dying. During the day, I was told, he had passed three quarts of urine, but none since 2 p. m. Passing the catheter, only two ounces were found in the bladder. Without premonitions he began to have convulsive twitchings of the facial muscles, rapidly passing into general convulsions. I found him in a comatose condition. All efforts to promote reaction failed, and at 9 p. m. he died.

In other disorders in which the kidneys may be affected, as pregnancy, disease or irritation of the central nervous system, and the essential fevers particularly, most valuable information, diagnostic, prognostic, and therapeutic, may be found by examination of the urine. It is not uncommon to find albuminuria in the malarial fevers, and chronic malarial toxæmia bears a well known etiological relation to Chronic Diffuse Nephritis. The anæmia, dropsy and other symptoms present in such cases may reasonably be attributed to the blood changes of malarial poisoning; urinalysis, however, will correct the mistake, by showing signs of diseased kidneys.

I present the next case as one of Chronic Diffuse Nephritis, dependent upon chronic malarial poisoning:

Mr. C., white, aged 23 years, consulted me in Feb'y. of this year for a persistent diarrhœa, which he said he could not cure. His family history was good; personally he was temperate. His general health was good until four years ago, when he began to have chills. Following this he has a history of chronic malarial poisoning, of four years duration; the attacks lasting from three to six months, with intervals of about three months. He has no headache, no cough, no dyspnœa, sleeps well, no loss of flesh, but is quite feeble, and has a poor appetite. He has occasional attacks of nausea and vomiting; lungs and heart normal; liver not enlarged; spleen, however, enlarged, can be felt below the ribs. Oedema of ankles, but no acites. Pulse 76; temperature 98.6. Skin has a peculiar grayish color, suggestive of grave trouble. Urine, daily quantity, 12 to 16 ounces; color, dark but not smoky; specific gravity, 1020; albumin, present in large quanties, 7 gm to the litre by measurement with Esbach's albuminometer; hyaline and granular casts, but no blood corpuscles under the microscope.

Treatment directed to elimination of excrementitious matters in the blood relieved his diarrhœa. He is now taking Flint's Tonic Chalybeate Tablets, with benefit.

The presence of albumin in the urine of individuals apparently in health, is sometimes accidentally discovered, notably during examinations for life insurance.

This albuminuria has attracted much attention during the last few years, though little is known of its nature. From its appearance in apparently healthy young adults at certain hours during the day, it has been variously called functional, physiological and cyclical albuminuria, or the albuminuria of adolescence. Exercise, diet, taking food, cold baths, mental strain, &c. seem to exercise an influence upon its presence, and it may possibly be associated with disorders of the blood making organs, renal circulation or nervous system. In this connection, I present the following cases:

J. S., white, age 24 years, height 5 ft. 10 1-2 in., weight 135 lbs. Habits temperate; no hereditary tendencies; heart, lungs, digestive and nervous functions normal; general health considered good. His urine passed during the examination had a specific gravity of 1028, albumin was present in a small amount, sugar was not present, nor were tube casts found, but crystals of oxalate of lime were abundant. He was requested to bring the first urine on rising next morning; also to measure the daily quantity. Three pints were passed in 24 hours. No albumin, no sugar, no tube casts were found in the early morning urine; while that passed at the office the second visit contained albumin. About twenty observations were made in this case with following results: Albumin absent in the early morning, a trace while at work. The daily average was less than 1 gm to the litre. Specific gravity 1022 to 1028, no sugar, and no tube casts were found. Taking food did not affect his urine. He never had any symptoms usual to renal disease. He passed then from observation until quite recently. Examination now of urine shows albumin, but no sugar, and no tube casts, while oxalate of lime crystals are still abundant; specific gravity 1032.

The next case was referred to me early in Feb'y; C. R., white, age 21 years, moulder by trade, height 5 ft. 8 in., weight 131 lbs., sallow complexion, stooping shoulders and flat chest. No hereditary or acquired disease. Heart, lungs, nervous system and digestion normal. He applied for life insurance, and, albumin being found in his urine, he was referred to me for further observation. He was requested to measure the urine passed in 24 hours, and bring specimens passed immediately on rising from bed, one hour after breakfast, one hour before and after dinner, and one hour before and after supper. Examinations of these yielded the following: after rising sp. gr. 1024, no albumin; after breakfast and on walking to work, sp. gr. 1028, albumin, a marked trace; before dinner while at work sp. gr. 1024, albumin, a trace; before supper after walking home sp. gr. 1028, albumin a marked trace; after supper, sp. gr. 1028, albumin, a trace. No sugar was present, nor were tube casts found in the entire quantity. The quantity of urine for 24 hours measured three pints. I have carried on a series of observations in this case, and have examined 75 specimens of urine, with following results. The daily quantity of urine is uniformly 45 to 55 ounces; specific gravity from 1022 to 1034, albumin is never found in early morning, it appears in marked traces after walking, in slight traces while working at his trade, while on two occasions, when he was requested to remain in bed, it was absent for the entire 24 hours. There was less than 1 gm of albumen to the litre, by measurement. Diet, and taking food had no effect on its presence. Tube casts have not been found, notwithstanding the most careful search; nor is sugar ever present. A careful watch for symptoms of Bright's Disease has been observed, and, with the exception of this albuminuria, this man is absolutely free from disease. He has taken for the past six weeks "Flints Tonic Chalybeate Tablets," with a gradual though not marked improvement in the albuminuria·

This albuminuria is claimed by some to be innocuous, by others to be pathological, therefore prejudicial, and these opinions of course exert their influence on the question of life insurance. With reference to the relation of this form of albuminuria to life insurance, I wrote to the medical directors of two of the companies for which I examine, Dr. A. DuBois, of the "Manhattan Life Insurance Company," of New York, and Dr. J. M. Keating, of the "Penn Mutual Life Insurance Company," of Philadelphia. Both say their companies will not entertain such risks. I embrace in full the opinion of Dr. Keating, as it expresses his views of the pathology of this disorder. He says: "There is no proof that albumin is ever found in the urine of healthy individuals; therefore, the term 'Functional Albuminuria' is not correct. But again, there is found in youths a trace of albumin at times, disappearing and then appearing, sometimes after meals, and especially after exercise, which is unaccompanied by casts, and which probably depends upon some curious disease of the blood, mal-assimilation, or on some form of enervation; I believe it is always the result of some pathological condition, more or less. Cases presenting this condition should not receive policies of insurance until this has entirely disappeared."

In regard to the diagnosis of these cases, attention to the following points will usually lead to a satisfactory conclusion. First, age, youth and young adult life. Second, sex, males most often affected. Third, general health usually good, ab-absence of symptoms suggestive of renal or cardiac disease.

Fourth, quantity of urine, normal. Fifth, specific gravity, high. Sixth, absence of tube casts—albumin with casts mean kidney disease. Eighth, accidental discovery, as during examination for life insurance.

The prognosis of this affection is as yet uncertain. The time since it was first observed is not long enough, nor the history of the cases so far collected of sufficient duration to draw positive conclusions. Some cases have been reported as passing finally into chronic renal disease; while very many gradually return to health. Extreme caution in the mode of living and tonic treatment are the only therapeutic indications so far recognized.

If, by this paper, I shall have been instrumental in arousing a deeper interest in the careful and frequent analysis of urine, and, by this means, aided in the employment of rational treatment in disease, I shall feel that my efforts have not been in vain.

